# Automated in-depth cerebral arterial labelling using cerebrovascular vasculature reframing and deep neural networks

**DOI:** 10.1038/s41598-023-30234-6

**Published:** 2023-02-24

**Authors:** Suk-Woo Hong, Ha-Na Song, Jong-Un Choi, Hwan-Ho Cho, In-Young Baek, Ji-Eun Lee, Yoon-Chul Kim, Darda Chung, Jong-Won Chung, Oh-Young Bang, Gyeong-Moon Kim, Hyun-Jin Park, David S. Liebeskind, Woo-Keun Seo

**Affiliations:** 1 Department of Neurology and Stroke Center, Samsung Medical Center, Sungkyunkwan University School of Medicine, Seoul, 06351 Korea; 2 Program in Brain Science, College of Natural Sciences, Seoul National University, Seoul, 08826 Korea; 3 Department of Digital Health, Samsung Advanced Institute for Health Sciences and Technology, Sungkyunkwan University School of Medicine, 81 Irwon-ro, Irwon-dong, Gangnam-gu, Seoul, 06351 Korea; 4 Department of Medical Artificial Intelligence, Konyang University, Daejeon, Korea; 5 Department of Electronic Electrical and Computer Engineering, Sungkyunkwan University, Suwon, 16419 Korea; 6 Center for Neuroscience Imaging Research, Institute for Basic Science (IBS), Suwon, 16419 Korea; 7 Division of Digital Healthcare, Yonsei University Mirae Campus, Wonju, 26493 Korea; 8 Department of Neurology and Comprehensive Stroke Center, UCLA, Los Angeles, CA USA

**Keywords:** Cerebrovascular disorders, Stroke

## Abstract

Identifying the cerebral arterial branches is essential for undertaking a computational approach to cerebrovascular imaging. However, the complexity and inter-individual differences involved in this process have not been thoroughly studied. We used machine learning to examine the anatomical profile of the cerebral arterial tree. The method is less sensitive to inter-subject and cohort-wise anatomical variations and exhibits robust performance with an unprecedented in-depth vessel range. We applied machine learning algorithms to disease-free healthy control subjects (*n* = 42), patients with stroke with intracranial atherosclerosis (ICAS) (*n* = 46), and patients with stroke mixed with the existing controls (*n* = 69). We trained and tested 70% and 30% of each study cohort, respectively, incorporating spatial coordinates and geometric vessel feature vectors. Cerebral arterial images were analyzed based on the ‘segmentation-stacking’ method using magnetic resonance angiography. We precisely classified the cerebral arteries across the exhaustive scope of vessel components using advanced geometric characterization, redefinition of vessel unit conception, and post-processing algorithms. We verified that the neural network ensemble, with multiple joint models as the combined predictor, classified all vessel component types independent of inter-subject variations in cerebral arterial anatomy. The validity of the categorization performance of the model was tested, considering the control, ICAS, and control-blended stroke cohorts, using the area under the receiver operating characteristic (ROC) curve and precision-recall curve. The classification accuracy rarely fell outside each image’s 90–99% scope, independent of cohort-dependent cerebrovascular structural variations. The classification ensemble was calibrated with high overall area rates under the ROC curve of 0.99–1.00 [0.97–1.00] in the test set across various study cohorts. Identifying an all-inclusive range of vessel components across controls, ICAS, and stroke patients, the accuracy rates of the prediction were: internal carotid arteries, 91–100%; middle cerebral arteries, 82–98%; anterior cerebral arteries, 88–100%; posterior cerebral arteries, 87–100%; and collections of superior, anterior inferior, and posterior inferior cerebellar arteries, 90–99% in the chunk-level classification. Using a voting algorithm on the queued classified vessel factors and anatomically post-processing the automatically classified results intensified quantitative prediction performance. We employed stochastic clustering and deep neural network ensembles. Ma-chine intelligence-assisted prediction of vessel structure allowed us to personalize quantitative predictions of various types of cerebral arterial structures, contributing to precise and efficient decisions regarding the cerebrovascular disease.

## Introduction

Defining the morphological nature of cerebral circulation and providing quantified information, so-called digitisation, are the indispensable hallmark of identifying pathogenic mechanisms, diagnosing disease, and determining the clinical relevance of cerebrovascular dysfunctions. Automated labelling of the major cerebral arterial branch is the first step for quantitatively analysing cerebral arterial morphology from cerebrovascular images. Characterising the considerable variations in complex intracranial vascular structures, half of which are usually located outside the Circle of Willis, has been challenging but is a crucial step in quantifying structural information of the cerebrovasculature^1^. Previous progressions include atlas-based artery identification and post-processing improvement using iterative region-growing territorial expansion and have suffered from practical limitations attributable to complexities and inter-individual variabilities of the cerebrovasculature covering only the major branches of the Circle of Willis for automated labelling^2–4^.

Here, we overcome the problems mentioned above, leveraging geometric features obtained from systematic time-of-flight magnetic resonance angiography (TOF MRA) with a deep neural network and advanced the performance of the models by reorganising vascular units for clinically disparate personalised cerebral vessel modules.

The strengths and unique nature of the investigation in this study reside in the validation of vessel unit restructuring and mathematical factor analysis in cerebrovasculature with the jointed assembly of machine learning models up to 62 cerebral branches. The study reveals that the prediction capacity of combined machine learning models fully automated and advanced the cerebral arterial labelling of queued vessel segments and minimised inter-individual anatomical variability confronted in clinical practice. The structural heterogeneity of control, stroke, and ICAS cerebrovasculature rarely undermined the prediction performance of the model ensemble.

This study aimed to segment the cerebrovascular arterial branches in a fully automatic manner. We focused on cerebral arterial circuits, systematising and validating neural network models to render them clinically pragmatic.

## Materials and methods

### Study design and subjects

In accordance with the primary purpose of this study which was the development of an automated cerebral arterial labelling algorithm, we used a pre-established cohort retrospectively. The study subjects were healthy controls, stroke patients, and stroke patients with intracranial atherosclerosis (ICAS) over 20 years (Fig. S1).

The control cohorts for the algorithm development were healthy subjects who visited the comprehensive health promotion centre at the Samsung Medical Center and who underwent MRA from January 1, 2013, to December 31, 2013, excluding those who had the following: (1) stroke including ischemic stroke, hemorrhagic stroke, and transient ischemic attack; (2) coronary artery or heart disease; (3) ICAS; (4) intracranial arterial anomalies corresponding to pathological conditions or variants of normal anatomy; (5) congenital morbidity including cerebral arterial hypoplasia; and (6) miscellaneous abnormal cases diagnosed by angiography.

Stroke patients for the external validation of the algorithm were selected from the Samsung Medical Center stroke registry (SMC stroke registry). This prospectively collected stroke registry recruited acute stroke patients seven days after stroke onset. Another specially selected stroke group was intended to provide clinical relevance to the algorithm from the patients who participated in The Intensive Statin Treatment in Acute Ischemic Stroke Patients with Intracranial Atherosclerosis—High-Resolution Magnetic Resonance Imaging (STAMINA-MRI) study^5^ with significant and symptomatic intracranial arterial stenosis of > 50% in the middle cerebral artery of the basilar artery.

Demographic data and vascular risk factors were collected from the medical records for controls and the stroke registry for the stroke cohort.

The Samsung Medical Center Institutional Review Board approved the study design (SMC-2021-04-072). This study was performed in accordance with the declaration of Helsinki. Informed consent was waived by the Samsung Medical Center Institutional Review Board for the control group because the study progressed in a retrospective manner, and we provided the clinical data and brain images in an anonymised form. Written informed consent was obtained from those enrolled in the SMC stroke registry.

### Imaging preparation

#### Magnetic resonance machine

The intracranial arteries were imaged using a 3.0 T Philips Achieva magnetic resonance imaging scanner (Philips Medical Systems) equipped with a 32-element phased-array receiver head coil.

#### Magnetic resonance sequence

This study used whole-brain three-dimensional (3D) MRA images with a TOF protocol collected from each participant. With an isotropic voxel size configured to 0.284 × 0.284 mm^3^, the parameters were as follows: echo time, 4.59 ms; repetition time, 22 ms; flip angle, 23°; RBW, 130 Hz/pixel; GRAPPA factor, 3; 32 reference lines.

#### Region growing

**Figure 1:**
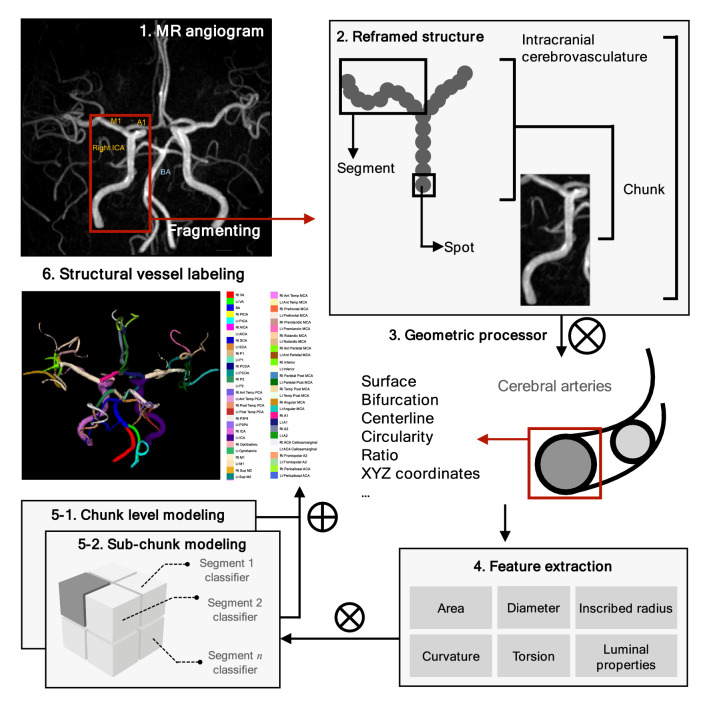
Flow schema describes the comprehensive preprocessing procedures and the geometric characterisation algorithms. Reconstruction encompasses vascular spots, segments, chunks, and branch units. Morphological and geometrcharacterisationion algorithms were used to process the vascular units automatically. A1, horizontal pre-communicating ACA; M1, sphenoidal middle cerebral artery; BA, basilar artery; ICA, internal carotid artery.

We used the raw input data format DICOM, which employs a TOF modality. Preprocessing procedures included anonymisation using DICOM Anonymizer Pro (Neologica, Montenotte, Italy) and region growing by an in-house vessel analyser program that created segmented brain angiography maps, converting them into the NII format. The internally developed vessel morphology pipelines then analysed and extracted brain vessel features to examine the cerebrovascular structure. Figure 1 provides a schema of the intravascular feature vector extraction process. Intelligent morphological surface, centerline, bifurcation, and airway sectional processing algorithms were used to characterise vascular spots, segments, chunks, and branches. The procedures were finalised upon providing multifaceted systematic dimensions of the geometrically modelled features.

#### Feature extraction

To specify feature modelling, the dissection of isosurfaces for vessel surface model generation was initially performed using the vascular modelling toolkit libraries^6–9^. The *z*-axis voxels were tailored to the isovoxel image scale using bicubic interpolation to minimise artefacts and rough image resampling on a regular planar grid^10–12^. A continuous 3D space was divided into myriad cells uniformly based on the respective vertices of their isosurfaces. The major arterial centerlines from the boundary surface of each cell were then extracted. At this stage, a set of spatial coordinates, whose nearest vertex among the vertices of the isosurface is homogeneously distributed, works as a unit cell. The framework determines the starting point and skeleton of the centerlines of the significant brain arteries from a lower slice of the vascular region for extraction. Vessel skeleton refinement strengthens the determination of the endpoints of the centerline. Specifically, (i) skeletonising the cerebrovascular region and surface, (ii) pruning the branch under a predetermined threshold, (iii) generating a linked list of a tree structure based on the refined skeletal structure, and (iv) specifying leaf nodes from the linked list determine the endpoints. The centerlines were extracted by tracking the boundary surfaces of the cells connecting the start and end points. Finally, the pipelines characterised numerous blood vessel feature vectors of compartmentalised groups based on a branch point of a centerline. Quantified vessel characteristics include the cerebral blood vessel cross-sectional area, maximally inscribed sphere radius, minimised and maximised diameter, maximum-minimum radius ratio, surface circumference, distortion, curvature, and (hydraulic) luminal circularity (Table S1).

Thus, the vascular morphological modelling pipelines precede the preprocessing. This leads to surface extraction and re-meshing, centerline and branch extraction, and centerline merging to obtain its derivative subfeatures. The finalised vessel features then support ensemble neural networks to detect structural brain anomalies. The feature vectors accompany subject-wise gold-standard ground truth vascular labels established by two board-certified neurologists with expertise in stroke imaging and angiography.

### Reframing hierarchical cerebrovasculature

In contrast to the conventional nomenclature employed in the clinical neurology field, approximately 4,000 detailed minuscule cerebral arterial segments, subdivided by our algorithm, required a dimensional reduction of the feature data for utilisation in neurovascular research. Therefore, we restructured the conventional concepts of the vascular unit system into four hierarchical levels (Fig. 1). ‘Spots’ are the most rudimentary units of cubic cells in the binarised 3D cerebral arterial tree with an interval of 0.2801 mm on the arterial centerline. Each spot, along with its morphological features, was reorganised and hybridised into segments according to continuity and ending between bifurcations. According to the vessel geometry upon bifurcation, feature vector extraction algorithms geometrically grouped segments into 62 branches. Branches corresponded to the conventional cerebral arterial nomenclature. The vessel branches composed of the vascular segments could be reconstructed according to clinically practical criteria of symmetry: anterior or posterior, basal or pial, and MCA, ACA, or PCA. The cerebrovasculature consisted of 20 types of vessel chunks. The appropriateness of this system was validated using uniform manifold approximation and projection (UMAP)^13,14^.

We provide a new nomenclature for the cerebral arteries (Table 1, Table S2).

**Table 1:** Vessel brancompartmentalisationion into a 20-chunk macrovessel framework composed of 62 major arterial segments delineated the whole cohort-wise performance range of vessel chunks and segments.

Vessel chunk (serial)	Accuracy (%)			Precision (%)			Recall (%)			F_1_ (%)		
	Control	Stroke	ICAS	Control	Stroke	ICAS	Control	Stroke	ICAS	Control	Stroke	ICAS
Right ICA	99	98	98	93	98	99	97	99	99	95	99	99
Left ICA	99	96	99	95	100	99	97	99	99	96	99	99
Right anterior basal MCA	95	90	92	84	96	95	82	97	96	83	97	96
Left anterior basal MCA	92	92	92	81	97	96	82	97	97	81	97	97
Right anterior pial MCA	98	96	99	97	99	99	97	99	100	97	99	99
Left anterior pial MCA	99	97	99	97	99	99	96	99	99	96	99	99
Right anterior basal ACA	91	87	89	72	95	96	68	95	94	70	95	95
Left anterior basal ACA	90	82	89	64	95	92	75	97	96	69	96	94
Right anterior pial ACA	91	90	95	70	95	97	66	96	96	68	96	96
Left anterior pial ACA	92	88	94	71	97	97	73	95	96	72	96	96
Right posterior VA	97	95	96	79	94	96	75	97	96	77	96	96
Left posterior VA	93	94	97	83	98	97	77	95	97	80	96	97
Right posterior basal PCA	97	91	97	80	97	98	84	98	97	82	97	97
Left posterior basal PCA	98	94	97	84	98	98	84	97	98	84	98	98
Right posterior pial PCA	87	87	93	69	97	97	65	86	96	67	91	96
Left posterior pial PCA	90	89	94	72	97	98	68	97	98	70	97	98
Right SCA, AICA, and PICA	96	94	95	84	93	96	90	98	98	87	96	97
Left SCA, AICA, and PICA	96	90	95	86	98	98	82	96	97	84	97	97
BA	96	93	95	85	97	96	80	97	95	82	97	96

^a^Each unit of major arterial segments was further condensed and tagged with identifiers composed of chunk and segment digits.

^b^Symmetrical properties of entire cerebrovascular branches were prefixed with ‘Right’ or ‘Left’; abbreviations are defined in the text.

^c^If the sample size was too small for inclusion in the test pool or there was only one type of branch within the chunk, the performance profile of the vessel branch was excluded.

ICA, internal carotid arteries; OA, ophthalmic arteries; ACHA, anterior choroidal arteries; VA, vertebral arteries; PICA, posterior inferior cerebellar arteries; AICA, anterior inferior cerebellar arteries; IAA, internal auditory arteries; SCA, superior cerebellar arteries; PCOA, posterior communicating arteries; PCA, posterior cerebral arteries; P1, pre-communicating PCA; P2, post-communicating PCA; P1P2, coalescence among P1 and P2; P3P4, mixture of quadrigeminal and calcarine PCA; PO, parieto-occipital arteries; PCALC, calcarine arteries; PPA, direct peduncular perforating arteries; HA, hippocampal arteries; PCAAT, anterior temporal PCA; PCAPT, posterior temporal PCA; PCALP, lateral posterior choroidal arteries; M1, sphenoidal middle cerebral artery; MCA, middle cerebral arteries; MCAS, superior division of MCA; MCAI, inferior division of MCA; MCALO, lateral orbitofrontal arteries; MCAPR, pre-Rolandic MCA; MCAR, Rolandic MCA; MCAAP, anterior parietal MCA; MCAPP, posterior parietal MCA; MCAA, angular MCA; MCAPT, posterior temporal MCA; MCAMT, middle temporal MCA; MCAAT, anterior temporal MCA; MCAPF, pre-frontal MCA; ACA, anterior cerebral arteries; A1, horizontal pre-communicating ACA; A2, vertical post-communicating pre-callosal ACA; A1A2, combination of A1 and A2; ACAMO, medial orbitofrontal ACA; A2F, frontopolar vertical post-communicating pre-callosal ACA; ACAC, callosomarginal ACA; ACAP, pericallosal ACA; BA, basilar artery; ACOA, anterior communicating artery.

### Classification method development

#### Two-step modelling

The primary objective of this study was the automatic segmentation and labelling of the cerebral vasculature using the conventional nomenclature (62 branches). To achieve this, we used a stepwise strategy: first allocating each spot of the whole brain to a specific chunk (step 1), and then allocating each spot of a single chunk to a specific branch (step 2). The supervised machine learning procedures initialised the allocation of each spot to a single chunk level using the multi-layer perceptron neural network^15–18^. Input features included the coordinates of each spot, cerebral blood vessel cross-sectional area, maximally inscribed sphere radius, minimised and maximised diameter, maximum-minimum radius ratio, surface circumference, distortion, curvature, and (hydraulic) luminal circularity. Subsequently, the final chunk assignment was performed by allocating each spot to a specific chunk (step 1). A similar process was repeated to allocate spots in a specific chunk to a single branch by supervised machine learning, and the subsequent voting process enhanced accuracy (step 2). The voting algorithm obtained profiles of classified vessel labels from spots and selected the most frequently appearing vessel label among vascular chunks and segments.

### Validation

An area under the receiver operating characteristic (AUROC) test was used to assess the internal and external validity and the chunk- and branch-level accuracy. First, we tested the performance of the algorithm in a set of independent stroke patients with ICAS. The structural dissimilarities in the cerebral arterial configuration between the healthy standard controls and those with stroke with ICAS were expected to indicate the clinical relevance of this algorithm in subjects with pathological conditions.

### Statistics

We performed groupwise $t$-statistics and ANOVA from the standpoints of feature vectors and vascular chunks to interpret between-group differences for statistical significance (Fig. S3). We visualised each group’s similarity profiles in a two-dimensional plane, excluding *t*-values with *p*-values > 0.001 as insignificant.

## Results

### Subject characteristics

Finally, we recruited 157 participants among the 203 screened subjects (42 / 50 controls, 46 / 77 stroke patients with ICAS, and 69 / 80 stroke patients; Fig. S1). Table 2 presents the demographics and distribution of the participants’ vascular risk factors. The training and test sets comprised 70% and 30%, respectively, of the healthy control group (*n* = 42). The group had a mean age of 58 years (SD, 10.1 years; Table 2) and was predominantly male (73.8%). A stroke group was included to validate the algorithm. The same training–test ratio policy was used for the rest of the study cohorts. The stroke-with-ICAS group comprised 46 stroke patients (58.7% male), with a mean age of 64.2 years (SD, 13.3). No data were excluded for any of the variables used in the training or testing sets. Stroke patients were older and more likely to smoke cigarettes and have diabetes, hyperlipidemia, hypertension, or derivative coronary artery diseases than those in the control group.

**Table 2:** Demographic and clinical features of the cohorts (*n* = 157).

Cases	Control (*n* = 42)	Stroke with ICAS (*n* = 46)	Stroke (*n* = 69)	*p*-value*	*p*-value^†^	*p*-value^‡^
Sex, male	31 (73.8)	27 (58.7)	77 (67.5)	0.138	< 0.001	0.433
Age, years	58 ± 10.1	64.2 ± 13.3	69.3 ± 12.8	0.018	< 0.001	< 0.001
Height, cm	166.3 ± 8.3	161.5 ± 9.7	163.4 ± 9	0.016	0.923	< 0.001
Weight, kg	67.4 ± 10.9	63.7 ± 12.5	65.8 ± 11.4	0.148	0.637	0.008
Hypertension	16 (38.1)	29 (63)	74 (66.7)	0.019	0.01	< 0.001
Diabetes	5 (11.9)	18 (42.9)	31 (27.9)	0.003	0.877	0.003
Hyperlipidaemia	11 (26.2)	18 (42.9)	56 (50.5)	0.201	0.006	< 0.001
^a^Current smoking	6 (16.7)	11 (23.9)	14 (20.3)	0.671	0.067	0.162
NIHSS		3.2 ± 4.2	2.8 ± 3.4		0.524	
TOAST^40^,					0.459	
Large artery atherosclerosis		34 (73.9)	12 (17.4)			
Cardioembolism		0 (0.0)	16 (23.2)			
Small vessel occlusion		0 (0.0)	16 (23.2)			
Other determined etiology		1 (2.2)	3 (4.3)			
Undetermined etiology		6 (13.0)	10 (14.5)			

^a^The intensity of smoking ranges from zero to two.

*Boldened if *p* < 0.05. *P-values* statisticalanalysedsed by homoscedastic two-sample t-test had two-tailed distribution parameters between the controls and stroke-with-ICAS patients.

^†^Homoscedastic two-sample t-test had two-tailed distribution parameters between the stroke-with-ICAS and stroke-only patients.

^‡^Homoscedastic two-sample t-test had two-tailed distribution parameters between the controls and stroke-only patients.

AF, atrial fibrillation; TOAST, Trial of Org 10,172 in Acute Stroke Treatment.

### Vascular component characteristics

Considering inter-subject hallmarks in the stepwise vessel identification process, the cerebrovascular images were evaluated under a vascular component profile containing 5016.4 ± 732.9 spots, 42.5 ± 4.2 segments, and 18.3 ± 0.8 chunks in controls; 4393.6 ± 1906.3 spots, 33.6 ± 11.7 segments, and 15.8 ± 4.3 chunks in patients with stroke with ICAS; and 4810.4 ± 1357.5 spots, 39 ± 7.6 segments, and 17.6 ± 1.9 chunks in patients with stroke. We considered the vessel unit distribution for each cohort to precisely analyse complex cerebral arterial trees (Fig. S5).

We mapped the distribution profile of spots across the control, stroke-with-ICAS, and stroke groups (Fig. S2). Regardless of study cohorts, concerning the vessel chunks, the most frequently appearing components were the right and left anterior pial MCA. They were followed by the right and left ICA, right anterior pial ACA, left posterior VA, and right and left posterior basal PCA with cohort-wise subtle fluctuations. Concerning the vessel segments, independent of the study groups, the four most frequently appearing vascular elements were the ICA and MCA angular branches, including the right and left branches. The following most frequent vascular segments included the pericallosal branch of the ACA and the posterior temporal branch of the PCA across the study groups, including the right and left branches.

### Testing the appropriateness of the reframed vascular structure

We evaluated the appropriateness of the reframed vascular elements by unsupervised dimensional reduction using the UMAP and visualised the cluster results in the planar space with colour mapping according to the 20 types of vessel chunks (Fig. S2-A, C, and E). The dimensionality reduction had nonlinear properties, and the global data structure was conserved. Each chunk’s lesional profile precisely depicted the characteristics of each vessel, and the embedding algorithms efficiently captured high dimensions. The qualitative observations showed that the control, stroke, and stroke-with-ICAS groups had similar overall accuracy in clustering.

Figures S2-B, D, and F depict the unsupervised clustering of the spots visualising each conventional vascular branch. Unlike in the chunk-level mapping, the branch-level clustering was insufficient to discriminate each branch.

### Step 1 modelling: chunk

**Figure 2:**
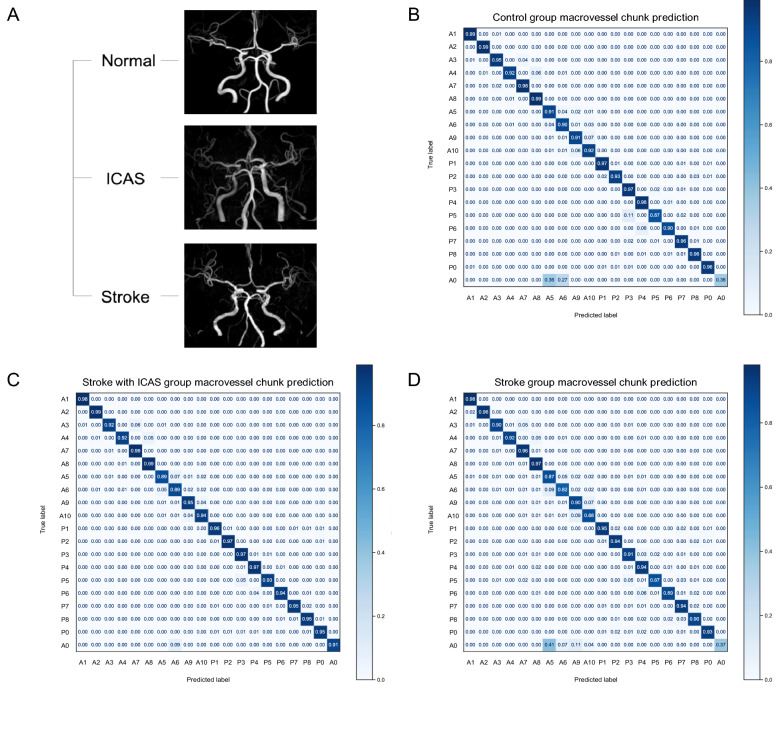
Prediction performance profiles of a 20-chunk macrovessel in the control, ICAS, and stroke group cohorts. (**A**) Examples of 3D cerebrovascular models of the disease-free healthy subjects, stroke-only patients, and stroke-with-ICAS patients. Macrovessel chunk prediction results of (**B**) control cohorts, (**C**) ICAS cohorts, and (**D**) stroke cohorts are shown. A0, anterior communicating artery; A1, branches of the right internal carotid arteries; A2, branches of the left internal carotid arteries; A3, basal branches of the right middle cerebral artery; A4, basal branches of the left middle cerebral artery; A5, basal branches of the right anterior cerebral artery; A6, basal branches of the left anterior cerebral artery; A7, pial branches of the right middle cerebral artery; A8, pial branches of the left middle cerebral artery; A9, pial branches of the right anterior cerebral artery; A10, pial branches of the left anterior cerebral artery; P0, basilar artery; P1, branches of the right vertebral artery; P2, branches of the left vertebral artery; P3, basal branches of the right posterior cerebral artery; P4, basal branches of the left posterior cerebral artery; P5, pial branches of the right posterior cerebral artery; P6, pial branches of the right posterior cerebral artery; P7, branches of the right superior cerebellar artery, anterior inferior cerebellar artery, and posterior inferior cerebellar artery; P8, branches of the left superior cerebellar artery, anterior inferior cerebellar artery, and posterior inferior cerebellar artery.

The model ensemble identified 20 chunks with 87–99% accuracy (Fig. 2), except in the left anterior basal ACA chunk in the stroke group (82%). The anterior communicating artery (ACOA; A0) showed cohort-wise fluctuating performance attributable to sample size constraints and significant anatomical variations (37–91%). The training and test sets included both epitomised and anomalous (Fig. 2A) 3D vascular component coordinates and derivative features at each spot vector as inputs. The controls, stroke cohort, and stroke-with-ICAS cohort gave similar results in that, except for A0, most of the vessel chunks (between 90 and 99%) were successfully classifiable. In the chunk prediction results of the control group (Fig. 2B), the majority of chunks showed 90–99% accuracy, except for the A0 (0.36) and P5 chunks (pial branches of the right posterior cerebral artery; 0.87). In the stroke group, only A5, A6, A10, P5, and P6 (basal branches of the right and left anterior cerebral artery, pial branches of the left anterior cerebral artery, and pial branches of the right and left posterior cerebral artery; 0.82–0.89) with A0 (0.37) fell outside the 90–99% range (Fig. 2D). In patients with ICAS (Fig. 2C), A5 and A6 (basal branches of the right and left anterior cerebral artery; 0.89) had an 89% accuracy and the rest of the branches, excluding ACOA, ranged within a 91–99% precision. When training both controls and stroke patients as a general group in chunk predictions (Fig. 2D), except for ACOA, the right and left anterior basal ACA and right and left posterior pial PCA chunks had an 82–89% performance. The rest showed unexceptional performances (90–98%) before the post-processing procedures.

With regard to sensitivity and specificity, the area under the curve (AUC) of the receiver operating characteristic (ROC) curve showed an overall value of 0.99–1.00 (0.97–1.00), and the precision-recall curve (PRC) was 0.992 (0.626–0.999) for the control, ICAS, and stroke cohorts. The algorithm’s AUC accuracy improved from 0.56–0.96 (95% CI 0.52–0.96; Table S3) to 0.62–0.98 (95% CI 0.56–0.98; Table S3) after applying the voting procedure, with statistical significance (*p* < 0.001) observed in all cases except for A5 and P5.

### Step 2 modelling: branch

**Figure 3:**
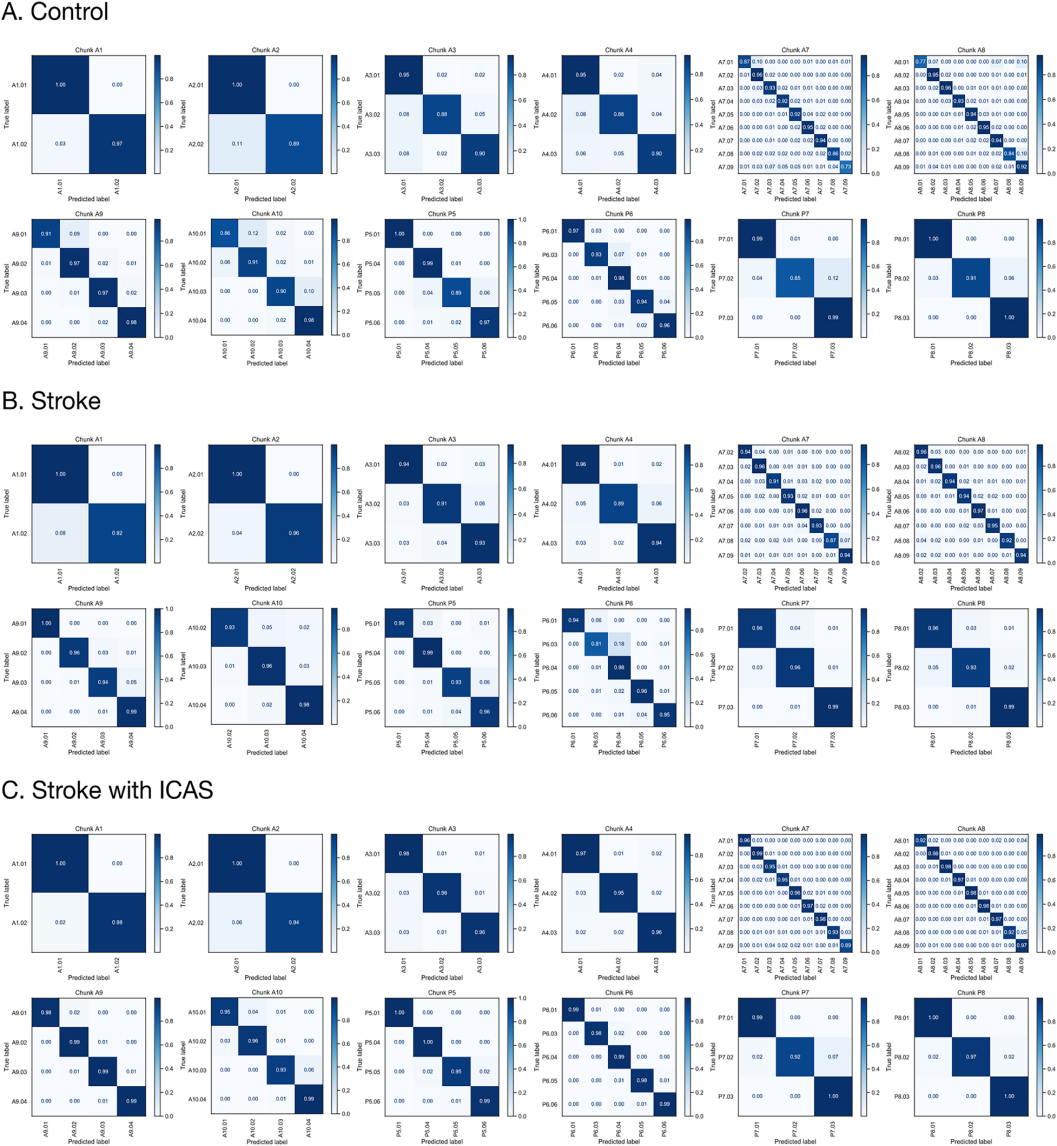
Prediction performance profiles of 62 major arterial branches in control, stroke-with-ICAS, and stroke group cohorts. The right and left posterior VA, BA, and ACOA chunks only include one significant type of arterial segment. The (**A**) control, (**B**) stroke, and (**C**) stroke-with-ICAS groups showed significant arterial segment classification capacity in the A0–A10 and P0–P8 chunks.

With regard to the branch-level classification of 62 branches in the controls, each spot had the following accuracy, except for a few exceptions: A1-A2: 89–100%, A3–A8: 73–96%, A9–A10: 86–98%, P5-P6: 89–100%, and P7-P8: 85–100% (Fig. 3, Table 1, Table S2). The control results showed that, except for A2.02, A3.02, A4.02, A7.01, A7.08, A7.09, A8.01, A8.08, A10.01, P5.05, and P7.02, the overall classification performance was roughly 90–99% (Table 1, Table S2). In the analysis of classification performance in the stroke cohorts, the following precision capacities were observed: A1-A2: 92–100%, A3-A8: 87–97%, A9-A10: 93–100%, P5-P6: 81–99%, and P7-P8: 93–99% (Fig. 3, Table 1, Table S2). Considering the deviations in the classification performance of the A4.02, A7.08, and P6.03 segments in the stroke group, the categorisation performance showed percentage values in the upper 90s (Table 1, Table S2). Further details of the classification performance are presented in Table 1 and Table S2. For segment-wise categorisation in ICAS patients, each type of segment had the following performance: A1-A2: 94–100%, A3-A8: 89–99%, A9-A10: 93–99%, P5-P6: 95–100%, and P7-P8: 92–100% (Fig. 3, Table 1, Table S2). Except for segment A7.09, the overall accuracy profile rarely fell below 90% (Table 1, Table S2).

Concerning sensitivity and specificity, the AUC-ROC showed an overall value of 0.99 (0.97–1.00), and the PRC was 0.992 (0.483–0.975) for the control, ICAS, and stroke cohorts scaled to macro-average and micro-average values (Fig. S6).

### Vascular morphological features according to the vascular risk factors

Fig. S3 presents differences in vascular morphological features among the control, stroke, and stroke-with-ICAS groups at each chunk. Notably, the phenomenon was conspicuous in the right ICA, left ICA, right anterior pial, and left anterior pial MCA chunks (Fig. 5). The ICAS group had similar propensities concerning characterised geometric features. However, we also discovered the following unforeseen heterogeneous chunks between the control-stroke-ICAS functional profiles (Fig. 5): anterior basal MCA, right anterior basal ACA, left anterior basal, right anterior pial ACA, and left anterior pial ACA.

### The profiles of geometric feature vectors weighted on deep neural networks

**Figure 4:**
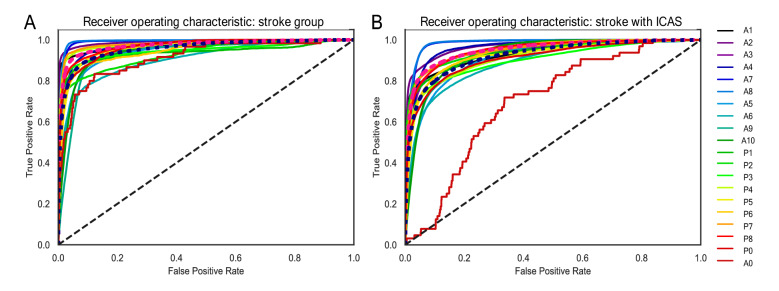
External validation via the receiver operating characteristic in the stroke-only and stroke-with-ICAS groups. Analysedsed the area under the receiver operating characteristic curve for the external validation of each spot’s deep neural network ensemble to a specific chunk for stroke patients. The employed model ensemble received training only with a control group tested and validated by (**A**) the stroke group and (**B**) the stroke-with-ICAS group.

The sensitivity and specificity results summarised by the AUROC revealed an overall micro-average performance of 0.97 and macro-average performance of 0.96 (Fig. 4A and Fig. S4) to discern stroke patients (*n* = 40) as external validation subjects, employing healthy subjects as the training set. When externally validated by the stroke-with-ICAS group (*n* = 46), the micro-average and macro-average performance values were 0.95 and 0.92, respectively (Fig. 4B and Fig. S4). For the external stroke patients, the AUROC performance ranged beyond the threshold of 0.95 for the entire vascular chunks except for A0, A5-6, A9, and P1-2, which spanned the 0.91–0.94 range. In contrast, in the external stroke-with-ICAS group, the AUROC scores of the vessel chunks A2, A4, A7–8, P2, and P7–8 exhibited values more extraordinary than the 0.95 capacity, leaving the rest of the chunks to bear performances between 0.90 and 0.94 (except for chunk A6 [0.89] and chunk A0 [0.70]).

## Discussion

This study demonstrated that patient-specific cerebral arterial profiles aid in the quantitative and in-depth labelling of cerebral arterial branches that is clinically useful^19–24^. We hypothesised that: (i) the appropriate combination of classical anatomical rules and computational prediction expedites the exhaustive classification of cerebral arteries; (ii) variations in vessel branches are detectable by employing neural networks. Accordingly, we designed a deep-learning algorithm that intelligently established in-depth vascular territories using strategies stemming from the systematic geometric characterisation of MRA data. The preprocessing modules uniquely reframed the entire cerebrovasculature into clinically redefined compartments: spots, segments, chunks, and branches.

A quantitative approach to investigating cerebrovasculature has shown limited success, and conventional approaches have used less precise quantitative methods to understand complex cerebrovascular structures with limited clinical relevance^25–27^. In previous attempts, vascular structures were overlooked, and distortion during thresholding was not adequately accounted for due to the shortage of appropriate geometric characterisation algorithms for examining small vessels. Additionally, these methods inadequately assessed residual variation, leading to overfitting, and thus their conclusions must be considered with caution. Furthermore, the cerebrovascular coverage of previous studies is lacking^27–29^, even excluding some vessel categories that were too complex to classify. The arterial geometry has also not been characterised; they are, therefore, vulnerable to normal variations.

MRA is one of the most widely used tools for assessing cerebral arterial disease. Using its template alignment, attempts have been made to label cerebral arteries limited to variations of those underlying the anterior circulation and those in clinical datasets or by bifurcations of interest, covering only roughly eight territorial frameworks using typical angiography of healthy subjects^3,30–33^. Therefore, we cannot claim to have included all of the components of the cerebrovasculature in our analysis. Several graph neural network studies have facilitated the construction of arterial morphometry, assuming that arterial traces are entirely intact and typically representative. In other words, their hypotheses are vulnerable to interpreting vascular abnormalities or variations such as vessel occlusion^4,25,26,34–36^. Thus, conventional anatomical rules are less appropriate for quantitatively understanding the cerebrovasculature.

### The role of neural networks in this study

**Figure 5:**
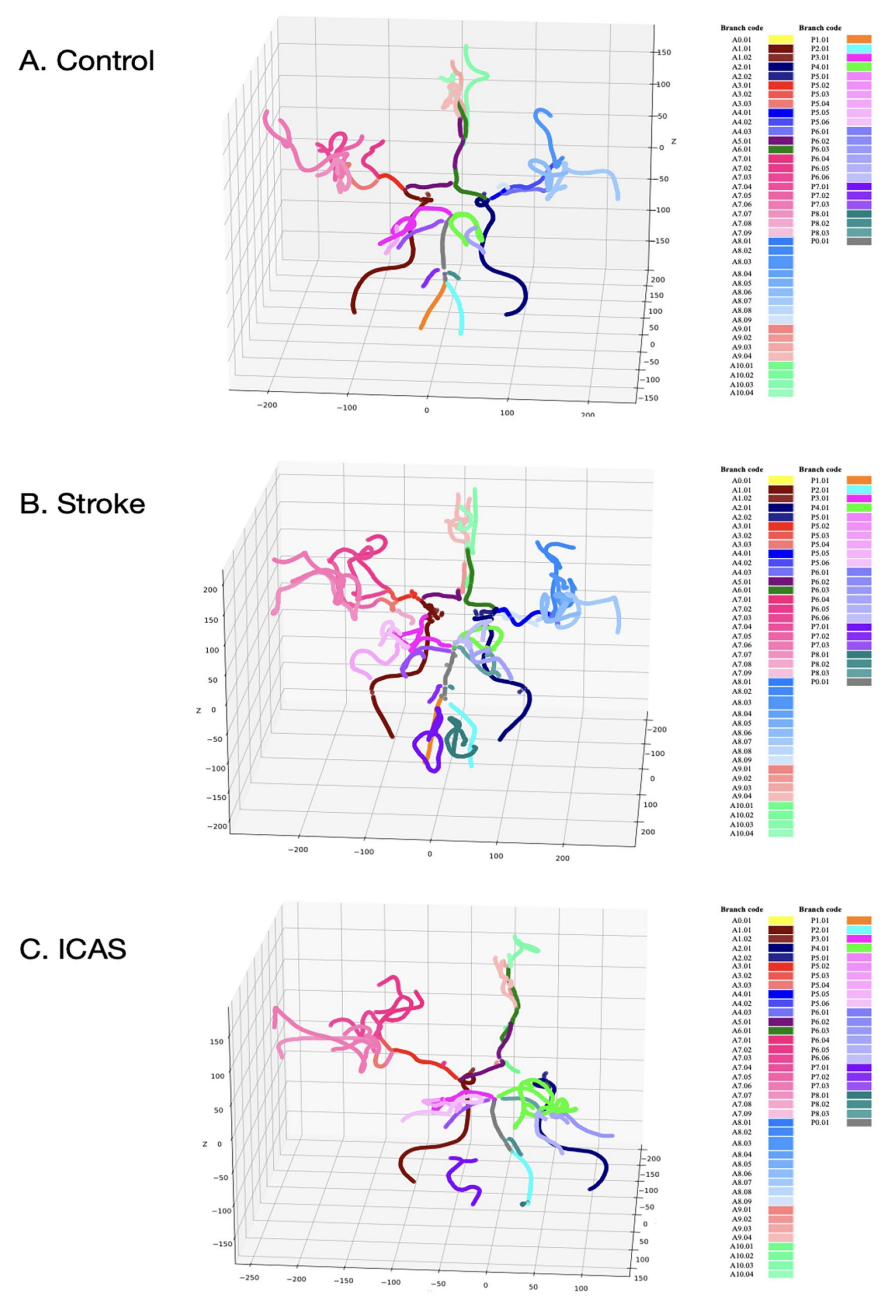
Clinical cases of the control, stroke and ICAS groups compared with predictively modeled vessel labels. Controls, stroke patients, and stroke-with-ICAS patients underwent colour labelling avisualisationion according to the branch within the cerebrovasculature. The algorithm consistently showed good performance for cerebral arterial branch identification among healthy controls, stroke patients, and stroke patients with ICAS. The structural abnormalities in patients with ICAS produced no significant adverse effects on identification performance.

The deep neural networks employed 3D coordinates and geometric vessel feature vectors (Fig. 5) derived from the reframed vascular fragments. We specifically tagged them into major arterial branches by employing segment-wise voting algorithms and orchestrated neural networks, creating 62 intelligently identifiable vessel territories. Implementing these algorithms does not require heavy graphics microprocessors, high resolution, advanced noise nullification, or a specific region of interest choice.

By stochastically clustering the classified vascular regions, we identified blood vessels by exploiting the conventional neuroanatomical branch nomenclature. Finally, we propose that morphological parameters such as diameter, roundness, and tortuosity strengthen characterisation vectors complementing variations under the Cartesian coordinates of angiographical categories. Therefore, reduced dependence on a Cartesian framework, owing to an overall uniform distribution of the contribution of geometric vascular features, lends this study a high level of robustness and reliability of performance.

Cerebral arterial labelling is not only a function of accuracy; therefore, targeted vessel chunks and segment ranges of the ensemble classifier cover should be exhaustive to fulfil clinical potential. Usually, pathological variations in the cerebral vasculature are expected to lower labelling performance. However, the multifaceted structural analysis among healthy controls, stroke patients, and stroke patients with ICAS revealed only a trivial difference in the performance of identifying each cerebral arterial branch (Fig. 5). Considering the consistent labelling performance of this algorithm, the distinct contribution of the weighting algorithm of each cubic cell consisting of a 3D vessel model is worthy of continued focus in future studies to improve accuracy. The prestructured models require an entire cerebral arterial label identification time of no more than a minute, unbridled by manual visual inspection bias and observer variabilities^37–39^. Therefore, this algorithm can be applied to investigate cerebrovascular morphological features in various settings, such as in a longitudinal study, assessment of drug effects, and between-group comparison.

### Paradigm-shifting vascular unit reframing

A mainstream strength of this study is the vascular framing using ‘chunk’, a critical element referring to a group of cerebrovascular elements sharing functional and anatomical similarities. As substantiated by the comparative analysis of UMAP assessment group-wise chunk-level feature vectors, chunk-level analysis is a rational categorisation method for territorialising cerebrovascular regions. Chunk discernment is a salient approach in a fundamental unit framework dedicated to the quantitative analysis of the cerebrovasculature and as an intermediate stage of vessel classification. Furthermore, this reframing reduces data dimension from about 5,000 spots to 20 chunks level, providing a valuable way of analysing and summarising the massive data into a valid format.

### Limitations and future directions

Several limitations exist. First, the results of this study have a limitation of generalizability. This study included participants with only an ischemic stroke rather than a hemorrhagic stroke. Thus, there are some potential challenges in discerning the various subtypes of the stroke to improve further the feasibility of the clinical application of neural network ensembles. In addition, all subjects were ethnically Korean. Considering the anatomical and pathological inter-racial differences in the intracranial cerebral vasculature, the performance of the model should be validated in different populations. Bespeaking generalizability and inter-institutional compatibility require additional external validation using images outside the Samsung Medical Center. Second, we acknowledge that the sample size at present is not sufficiently large; however, based on patient-specific profiles, we have provided the potential for finding additional novel brain frailty biomarkers. Third, our results indicate that neural networks effectively identify the cerebral arterial segment. However, a few structure-ambiguous vascular segments suffer from unsatisfactory performance complemented via segment-wise voting post-processing. We hypothesise that the enigmatic nature of some small vessel segments predisposes them to idiosyncratic performances. The geometric features of the developed redefined vascular units provide groundbreaking opportunities to interpret mixed vascular territories intelligently. However, covering various subjects with reproducible iterative measurements and theorising optimised feature combinations remains an important scientific question. Another limitation of this study is that we only used TOF MR angiographic images. MRA does not, per se, provide authentic structural images but includes hemodynamic information. Therefore, modifications are mandatory if we apply the model developed in this study to other imaging modalities, such as digital subtraction angiography or computed tomographic angiography.

Our system utilises segment-wise voting algorithms, anatomical post-processing, paradigm-shifting vascular unit reframing, and robust systematic geometric feature characterisation for clinical usability. The model performance was further synchronised and engineered using anatomical rule-assisted post-processing complementation. Exhaustive categorisation resulted in segmenting the 20 vascular chunks, which were narrowed down and further classified into small vessel segment compartments composed of spot cells. The unlimited applicability of orchestrated neural networks to non-matched feature vector data provides patient-specific profiles, facilitating successful clinical interpretations, the potential prognosis of brain debility progression, and post-stroke treatment management.

## Conclusions

In conclusion, we have pioneered the in-depth labelling cerebral arterial segments by scrutinising neural network ensembles via cerebrovascular structural reframing. By systematically analysing diverse cohorts, our results demonstrate that this technique is feasible and robust in profiling vessel-specific labelling.

## Supplementary Information

Supplementary Information.

## Data Availability

According to the Korean governmental policy and health security policy of the data sharing committee of the Samsung Medical Center, all clinical information and brain image data are limitedly available through formal approval procedures upon requests to validated investigators. Further requests and inquiries are available to corresponding author (W.-K. Seo).

## Abbreviations

ICA: Internal carotid arteries
OA: Ophthalmic arteries
ACHA: Anterior choroidal arteries
VA: Ver-tebral arteries
PICA: Posterior inferior cerebellar arteries
AICA: Anterior inferior cerebellar arter-ies
IAA: Internal auditory arteries
SCA: Superior cerebellar arteries
PCOA: Posterior communi-cating arteries
PCA: Posterior cerebral arteries
P1: Pre-communicating PCA
P2: Post-communicating PCA
P1P2: Merging of P1 and P2
P3P4: Mixture of quadrigeminal and calcarine PCA
PO: Parieto-occipital arteries
PCALC: Calcarine arteries
PPA: Direct peduncular perforating arteries
HA: Hippocampal arteries
PCAAT: Anterior temporal PCA
PCAPT: Posterior temporal PCA
PCALP: Lateral posterior choroidal arteries
M1: Sphenoidal middle cerebral artery
MCA: Middle cerebral arteries
MCAS: Superior division of MCA
MCAI: Inferior division of MCA
MCALO: Lateral orbitofrontal arteries
MCAPR: Pre-Rolandic MCA
MCAR: Rolandic MCA
MCAAP: Anterior parietal MCA
MCAPP: Posterior parietal MCA
MCAA: Angular MCA
MCAPT: Posterior temporal MCA
MCAMT: Middle temporal MCA
MCAAT: Anterior temporal MCA
MCAPF: Pre-frontal MCA
ACA: Anterior cerebral arteries
A1: Horizontal pre-communicating ACA
A2: Vertical post-communicating pre-callosal ACA
A1A2: Combination of A1 and A2
ACAMO: Medial orbitofrontal ACA
A2F: Frontopolar vertical post-communicating pre-callosal ACA
ACAC: Callosomarginal ACA
ACAP: Pericallosal ACA
BA: Basilar artery
ACOA: Anterior communicating artery
