# Supplementary material for: Automated in-depth cerebral arterial labelling using cerebrovascular vasculature reframing and deep neural networks
Source: Sci Rep. 2023 Feb 24;13:3255. doi: 10.1038/s41598-023-30234-6 (PMC9957982; doi:10.1038/s41598-023-30234-6)
Supplement: Supplementary file 1 — Supplementary Information. [file 41598_2023_30234_MOESM1_ESM.docx]

**Supplementary Online Only Material**

**Deep neural networks disambiguate cerebrovascular complexities arising from structural variations: cerebrovascular structural reframing and automated in-depth labeling**

**Contents**

**Supplementary Figure 1.** Inclusion flow chart of study cohorts.

**Supplementary Figure 2.** Results of stochastic chunk- and segment-based cerebrovascular clustering analysis.

**Supplementary Figure 3.** Results of ANOVA F-statistics with analysis of the vascular characterization vectors and macrovascular chunks.

**Supplementary Figure 4.** The contribution profiles of geometric feature vectors weighted when training and employing deep neural networks.

**Supplementary Figure 5.** Cohort-wise vessel count profiles scaled in vascular chunk and segment.

**Supplementary Figure 6.** Cohort-wise receiver operating characteristic and precision-recall curve scaled in vessel chunk.

**Supplementary Table 1.** Evaluation method description of intracranial vasculature features.

**Supplementary Table 2.** Vessel branch compartmentalization into a 20-chunk macrovessel framework composed of 62 major arterial segments and delineated whole cohort-wise performance range of vessel chunks and segments.

**Supplementary Table 3.** Comparison of the chunk-level discrimination performance between the models with and without the voting procedure.

**Supplementary Figure 1. Inclusion flow chart of study cohorts.**

**Supplementary Figure 2. Results of stochastic chunk- and segment-based cerebrovascular clustering analysis.**

Clustering mapping results are presented by (A) chunks in the control group, (B) segments in the control group, (C) chunks in the ICAS group, (D) segments in the ICAS group, (E) chunks in TOAST levels 1–3, (F) segments in TOAST levels 1–3, (G) chunks in the stroke group, and (H) segments in the stroke group.

A0, anterior communicating artery; A1, branches of the right internal carotid arteries; A2, branches of the left internal carotid arteries; A3, basal branches of the right middle cerebral artery; A4, basal branches of the left middle cerebral artery; A5, basal branches of the right anterior cerebral artery; A6, basal branches of the left anterior cerebral artery; A7, pial branches of the right middle cerebral artery; A8, pial branches of the left middle cerebral artery; A9, pial branches of the right anterior cerebral artery; A10, pial branches of the left anterior cerebral artery; P0, basilar artery; P1, branches of the right vertebral artery; P2, branches of the left vertebral artery; P3, basal branches of the right posterior cerebral artery; P4, basal branches of the left posterior cerebral artery; P5, pial branches of the right posterior cerebral artery; P6, pial branches of the right posterior cerebral artery; P7, branches of the right superior cerebellar artery, anterior inferior cerebellar artery, and posterior inferior cerebellar artery; P8, branches of the left superior cerebellar artery, anterior inferior cerebellar artery, and posterior inferior cerebellar artery

**Supplementary Figure 3. Results of ANOVA F-statistics with analysis of the vascular characterization vectors and macrovascular chunks.**

The ANOVA values with p-values greater than 0.001 were considered insignificant and marginalized as zero. The results of the ANOVA compare the control, stroke, and stroke-with-ICAS groups.

A0, anterior communicating artery; A1, branches of the right internal carotid arteries; A2, branches of the left internal carotid arteries; A3, basal branches of the right middle cerebral artery; A4, basal branches of the left middle cerebral artery; A5, basal branches of the right anterior cerebral artery; A6, basal branches of the left anterior cerebral artery; A7, pial branches of the right middle cerebral artery; A8, pial branches of the left middle cerebral artery; A9, pial branches of the right anterior cerebral artery; A10, pial branches of the left anterior cerebral artery; P0, basilar artery; P1, branches of the right vertebral artery; P2, branches of the left vertebral artery; P3, basal branches of the right posterior cerebral artery; P4, basal branches of the left posterior cerebral artery; P5, pial branches of the right posterior cerebral artery; P6, pial branches of the right posterior cerebral artery; P7, branches of the right superior cerebellar artery, anterior inferior cerebellar artery, and posterior inferior cerebellar artery; P8, branches of the left superior cerebellar artery, anterior inferior cerebellar artery, and posterior inferior cerebellar artery

**Supplementary Figure 4. The contribution profiles of geometric feature vectors weighted when training and employing deep neural networks.**

The contribution of Cartesian coordinates and geometric features when using the model ensemble trained only by a control group tested and validated by a stroke-with-ICAS group with a hierarchy.

**Supplementary Figure 5. Cohort-wise vessel count profiles scaled in vascular chunk and segment.**

The results show the distribution of vessel components scaled in chunks and segments investigating dominant vessel components in each cohort.

A0, anterior communicating artery; A1, branches of the right internal carotid arteries; A2, branches of the left internal carotid arteries; A3, basal branches of the right middle cerebral artery; A4, basal branches of the left middle cerebral artery; A5, basal branches of the right anterior cerebral artery; A6, basal branches of the left anterior cerebral artery; A7, pial branches of the right middle cerebral artery; A8, pial branches of the left middle cerebral artery; A9, pial branches of the right anterior cerebral artery; A10, pial branches of the left anterior cerebral artery; P0, basilar artery; P1, branches of the right vertebral artery; P2, branches of the left vertebral artery; P3, basal branches of the right posterior cerebral artery; P4, basal branches of the left posterior cerebral artery; P5, pial branches of the right posterior cerebral artery; P6, pial branches of the right posterior cerebral artery; P7, branches of the right superior cerebellar artery, anterior inferior cerebellar artery, and posterior inferior cerebellar artery; P8, branches of the left superior cerebellar artery, anterior inferior cerebellar artery, and posterior inferior cerebellar artery

**Supplementary Figure 6. Cohort-wise receiver operating characteristic and precision-recall curve scaled in vessel chunk.**

The results show the receiver operating characteristic and precision-recall curves of vessel components scaled in chunks investigating classification performance validation in each cohort. Control, stroke, and general group each refers to disease-free healthy subjects, stroke patients, and mixture of control and stroke cohort.

**Supplementary Table 1. Evaluation method description of intracranial vasculature features.**

| **Feature vectors** | **Evaluation** |
| --- | --- |
| X, Y, and Z-coordinate | 3 dimensional cubic cell coordinate of X, Y, and Z axis in cartesian framework |
| Max inscribed sphere R | Insphere which is sphere contained within the vessel segment polyhedron tangent to surfaces of it |
| Hydraulic luminal diameter | Hydraulic diamter evaluated by ratio cross-sectional area of the flow to perimeter of the arterial segment channel |
| Minimum and maximum diameter | Maximum and minimum diameter calculated among the diameters within the vascular segment |
| Area | Cross sectional areas of vessel segment |
| Perimeter | Continuous line of boundary of geometric vessel channel |
| Luminal circularity | Roundness of lumen by (4π**A*)/*P*^2^ |
| Minimum to maximum diameter ratio | Ratio of maximized to minimized diameter calculated among the diameters within the vascular segment |
| Curvature | Degree of deviation of straight cylindric arterial channel |
| Torsion | Tortuosity parameter evaluated by path length, wavelength, and radius of curvature of an arterial segment |

*A*, area; *P*, perimeter

**Supplementary Table 2. Vessel branch compartmentalization into a 20-chunk macrovessel framework composed of 62 major arterial segments and delineated whole cohort-wise performance range of vessel chunks and segments.**

| **Major arterial segment;**  **vessel branch (serial)** | **Accuracy (%)** | | | **Precision (%)** | | | **Recall (%)** | | | **F_1_ (%)** | | |
| --- | --- | --- | --- | --- | --- | --- | --- | --- | --- | --- | --- | --- |
|  | **Control** | **Stroke** | **ICAS** | **Control** | **Stroke** | **ICAS** | **Control** | **Stroke** | **ICAS** | **Control** | **Stroke** | **ICAS** |
| Right ICA^b^ (A1.01^a^)  Right OA (A1.02) | 100  97 | 100  92 | 100  98 | 100  97 | 100  95 | 100  95 | 100  95 | 100  98 | 100  98 | 100  96 | 100  97 | 100  97 |
| Left ICA (A2.01)  Left OA (A2.02) | 100  89 | 100  96 | 100  94 | 100  97 | 100  99 | 100  99 | 100  94 | 100  97 | 100  97 | 100  95 | 100  98 | 100  98 |
| Right M1 (A3.01)  Right MCAS (A3.02)  Right MCAI (A3.03) | 95  88  90 | 94  91  93 | 98  96  96 | 89  70  86 | 98  97  97 | 99  95  95 | 84  79  85 | 98  96  98 | 98  95  98 | 87  74  86 | 98  96  97 | 98  95  97 |
| Left M1 (A4.01)  Left MCAS (A4.02)  Left MCAI (A4.03) | 95  88  90 | 96  89  94 | 97  95  96 | 89  74  85 | 98  95  98 | 98  93  96 | 90  80  81 | 98  97  97 | 98  95  97 | 90  77  83 | 98  96  98 | 98  94  97 |
| Right MCALO (A7.01)  Right MCAPR (A7.02)  Right MCAR (A7.03)  Right MCAAP (A7.04)  Right MCAPP (A7.05)  Right MCAA (A7.06)  Right MCAPT (A7.07)  Right MCAMT (A7.08)  Right MCAAT (A7.09) | 87  96  93  92  92  95  94  86  73 | 94  96  91  93  96  93  87  94 | 96  99  95  95  96  97  98  93  89 | 72  68  82  52  70 | 97  96  96  96  98  93  97  96 | 99  98  96  97  97  98  96  92  87 | 79  74  77  72  74 | 95  97  95  96  96  97  95  96 | 96  97  98  97  95  97  97  95  94 | 75  71  79  60  72 | 96  97  96  96  97  95  96  96 | 98  98  97  97  96  98  96  94  90 |
| Left MCALO (A8.01)  Left MCAPR (A8.02)  Left MCAR (A8.03)  Left MCAAP (A8.04)  Left MCAPP (A8.05)  Left MCAA (A8.06)  Left MCAPT (A8.07)  Left MCAMT (A8.08)  Left MCAAT (A8.09) | 77  95  96  93  94  95  94  84  92 | 96  96  94  94  97  95  92  94 | 92  98  98  97  98  98  97  92  97 | 67  62  61  56  85  57  63 | 96  95  94  92  97  93  95  98 | 95  98  98  96  93  98  94  98  97 | 71  66  50  53  79  67  70 | 95  95  92  93  97  95  94  95 | 88  99  96  91  96  97  97  99  97 | 69  64  55  55  82  61  66 | 95  95  93  93  97  94  94  96 | 91  98  97  93  95  98  95  99  97 |
| Right A1A2 (A5.01) |  | | |  | | |  | | |  | | |
| Left A1A2 (A6.01) |  | | |  | | |  | | |  | | |
| Right ACAMO (A9.01)  Right A2F (A9.02)  Right ACAC (A9.03)  Right ACAP (A9.04) | 91  97  97  98 | 100  96  94  99 | 98  99  99  99 | 85  81  96 | 99  98  99 | 89  99  99  100 | 75  89  92 | 98  99  99 | 94  99  99  99 | 79  85  94 | 99  99  99 | 91  99  99  100 |
| Left ACAMO (A10.01)  Left A2F (A10.02)  Left ACAC (A10.03)  Left ACAP (A10.04) | 86  91  90  98 | 93  96  98 | 95  96  93  99 | 77  86  90 | 94  98  98 | 96  98  99  99 | 69  80  95 | 98  96  99 | 91  98  98  99 | 73  83  93 | 96  97  98 | 93  98  98  99 |
| Right VA (P1.01) |  | | |  | | |  | | |  | | |
| Left VA (P2.01) |  | | |  | | |  | | |  | | |
| Right P1P2P3P4 (P3.01) |  | | |  | | |  | | |  | | |
| Left P1P2P3P4 (P4.01) |  | | |  | | |  | | |  | | |
| Right PCOA (P5.01)  Right HA^c^ (P5.02)  Right PCAAT (P5.03)  Right PCAPT (P5.04)  Right PO (P5.05)  Right PCALC (P5.06) | 100  99  89  97 | 96  99  93  96 | 100  100  95  99 | 91 | 73 | 92 | 99 | 99 | 100 | 95 | 84 | 96 |
| Left PCOA (P6.01)  Left HA (P6.02)  Left PCAAT (P6.03)  Left PCAPT (P6.04)  Left PO (P6.05)  Left PCALC (P6.06) | 97  93  98  94  96 | 94  81  98  96  95 | 99  98  99  98  99 | 98  73  85  70  92 | 100  92  99  98  99 | 100  94  98  99  99 | 97  71  94  57  81 | 95  94  99  97  99 | 96  96  98  98  99 | 98  72  89  63  86 | 97  93  99  98  99 | 98  95  98  98  99 |
| Right PICA (P7.01)  Right AICA (P7.02)  Right SCA (P7.03) | 99  85  99 | 96  96  99 | 99  92  100 | 79  91  95 | 99  99  100 | 100  99  100 | 81  85  99 | 99  98  100 | 99  99  100 | 80  88  97 | 99  98  100 | 100  99  100 |
| Left PICA (P8.01)  Left AICA (P8.02)  Left SCA (P8.03) | 100  91  100 | 96  93  99 | 100  97  100 | 91  82  94 | 99  93  99 | 100  96  99 | 80  86  97 | 96  97  100 | 99  96  100 | 85  84  96 | 97  95  100 | 99  96  99 |
| BA (P0.00) |  | | |  | | |  | | |  | | |
| ACOA (A0.00) |  | | |  | | |  | | |  | | |

^a^Symmetrical properties of entire cerebrovascular branches were prefixed with ‘Right’ or ‘Left’; abbreviations are defined in the text.

^b^Each unit of major arterial segments was further condensed and tagged with identifiers composed of chunk and segment digits.

^c^If the sample size was too small for inclusion in the test pool or there was only one type of branch within the chunk, the performance profile of the vessel branch was excluded.

ICA, internal carotid arteries; O.A., ophthalmic arteries; ACHA, anterior choroidal arteries; V.A., vertebral arteries; PICA, posterior inferior cerebellar arteries; AICA, anterior inferior cerebellar arteries; IAA, internal auditory arteries; SCA, superior cerebellar arteries; PCOA, posterior communicating arteries; PCA, posterior cerebral arteries; P1, pre-communicating PCA; P2, post-communicating PCA; P1P2, coalescence among P1 and P2; P3P4, mixture of quadrigeminal and calcarine PCA; P.O., parieto-occipital arteries; PCALC, calcarine arteries; PPA, direct peduncular perforating arteries; H.A., hippocampal arteries; PCAAT, anterior temporal PCA; PCAPT, posterior temporal PCA; PCALP, lateral posterior choroidal arteries; M1, sphenoidal middle cerebral artery; MCA, middle cerebral arteries; MCAS, superior division of MCA; MCAI, inferior division of MCA; MCALO, lateral orbitofrontal arteries; MCAPR, pre-Rolandic MCA; MCAR, Rolandic MCA; MCAAP, anterior parietal MCA; MCAPP, posterior parietal MCA; MCAA, angular MCA; MCAPT, posterior temporal MCA; MCAMT, middle temporal MCA; MCAAT, anterior temporal MCA; MCAPF, pre-frontal MCA; ACA, anterior cerebral arteries; A1, horizontal pre-communicating ACA; A2, vertical post-communicating pre-callosal ACA; A1A2, combination of A1 and A2; ACAMO, medial orbitofrontal ACA; A2F, frontopolar vertical post-communicating pre-callosal ACA; ACAC, callosomarginal ACA; ACAP, pericallosal ACA; B.A., basilar artery; ACOA, anterior communicating artery

**Supplementary Table 3. Comparison of the chunk-level discrimination performance between the models with and without the voting procedure.**

| Chunk | | Without voting | With voting | *p*-value |
| --- | --- | --- | --- | --- |
| Number | Name | AUC (95% CI) | AUC (95% CI) |  |
| A0 | ACOA | 0.56 (0.52 - 0.60) | 0.62 (0.56 - 0.67) | 0.015 |
| A1 | Right ICA | 0.90 (0.90 - 0.91) | 0.94 (0.94 - 0.94) | <0.001 |
| A2 | Left ICA | 0.92 (0.92 - 0.92) | 0.96 (0.95 - 0.96) | <0.001 |
| A3 | Right anterior basal MCA | 0.81 (0.81 - 0.82) | 0.85 (0.84 - 0.86) | <0.001 |
| A4 | Left anterior basal MCA | 0.81 (0.80 - 0.81) | 0.85 (0.85 - 0.86) | <0.001 |
| A7 | Right anterior pial MCA | 0.69 (0.69 - 0.70) | 0.70 (0.69 - 0.70) | 0.644 |
| A8 | Left anterior pial MCA | 0.72 (0.71 - 0.72) | 0.75 (0.75 - 0.76) | <0.001 |
| A5 | Right anterior basal ACA | 0.95 (0.95 - 0.95) | 0.97 (0.97 - 0.97) | <0.001 |
| A6 | Left anterior basal ACA | 0.96 (0.96 - 0.96) | 0.98 (0.98 - 0.98) | <0.001 |
| A9 | Right anterior pial ACA | 0.73 (0.73 - 0.74) | 0.76 (0.76 - 0.77) | <0.001 |
| A10 | Left anterior pial ACA | 0.75 (0.74 - 0.75) | 0.77 (0.77 - 0.78) | <0.001 |
| P1 | Right posterior VA | 0.81 (0.80 - 0.81) | 0.85 (0.84 - 0.85) | <0.001 |
| P2 | Left posterior VA | 0.81 (0.80 - 0.82) | 0.84 (0.83 - 0.85) | <0.001 |
| P3 | Right posterior basal PCA | 0.81 (0.81 - 0.82) | 0.83 (0.83 - 0.84) | <0.001 |
| P4 | Left posterior basal PCA | 0.86 (0.86 - 0.86) | 0.90 (0.90 - 0.91) | <0.001 |
| P5 | Right posterior pial PCA | 0.90 (0.89 - 0.90) | 0.95 (0.94 - 0.95) | <0.001 |
| P6 | Left posterior pial PCA | 0.70 (0.68 - 0.71) | 0.71 (0.70 - 0.72) | 0.064 |
| P7 | Right SCA, AICA, and PICA | 0.73 (0.72 - 0.75) | 0.71 (0.70 - 0.72) | <0.001 |
| P8 | Left SCA, AICA, and PICA | 0.86 (0.86 - 0.87) | 0.90 (0.89 - 0.90) | <0.001 |
| P0 | BA | 0.80 (0.80 - 0.81) | 0.86 (0.86 - 0.87) | <0.001 |

Excluding the voting area under the curve (AUC) allows for each spot to be allocated correctly into a specific chunk using only the multi-layer perceptron (MLP) neural network. In contrast, AUC with voting allows for the same possibility using a hybridized model composed of the MLP neural network and a voting procedure. ROC curve analysis comparing voting and non-voting was performed.
